# The repurposed anthelmintic mebendazole in combination with trametinib suppresses refractory NRAS^Q61K^ melanoma

**DOI:** 10.18632/oncotarget.14990

**Published:** 2017-02-02

**Authors:** Cynthia M. Simbulan-Rosenthal, Sivanesan Dakshanamurthy, Anirudh Gaur, You-Shin Chen, Hong-Bin Fang, Maryam Abdussamad, Hengbo Zhou, John Zapas, Valerie Calvert, Emanuel F. Petricoin, Michael B. Atkins, Stephen W. Byers, Dean S. Rosenthal

**Affiliations:** ^1^ Department of Biochemistry and Molecular & Cellular Biology, Georgetown University Medical Center, Washington, DC, USA; ^2^ Department of Oncology, Lombardi Comprehensive Cancer Center, Georgetown University Medical Center, Washington, DC, USA; ^3^ Department of Biostatistics, Bioinformatics, and Biomathematics, Georgetown University Medical Center, Washington, DC, USA; ^4^ MedStar Franklin Square Medical Center, Baltimore, MD, USA; ^5^ Center for Applied Proteomics and Molecular Medicine, George Mason University, Manassas, VA, USA

**Keywords:** mebendazole, drug repurposing, melanoma, ERK pathway, BRAF

## Abstract

Structure-based drug repositioning in addition to random chemical screening is now a viable route to rapid drug development. Proteochemometric computational methods coupled with kinase assays showed that mebendazole (MBZ) binds and inhibits kinases important in cancer, especially both BRAF^WT^ and BRAF^V600E^. We find that MBZ synergizes with the MEK inhibitor trametinib to inhibit growth of BRAF^WT^-NRAS^Q61K^ melanoma cells in culture and in xenografts, and markedly decreased MEK and ERK phosphorylation. Reverse Phase Protein Array (RPPA) and immunoblot analyses show that both trametinib and MBZ inhibit the MAPK pathway, and cluster analysis revealed a protein cluster showing strong MBZ+trametinib - inhibited phosphorylation of MEK and ERK within 10 minutes, and its direct and indirect downstream targets related to stress response and translation, including ElK1 and RSKs within 30 minutes. Downstream ERK targets for cell cycle, including cMYC, were down-regulated, consistent with S- phase suppression by MBZ+trametinib, while apoptosis markers, including cleaved caspase-3, cleaved PARP and a sub-G1 population, were all increased with time. These data suggest that MBZ, a well-tolerated off-patent approved drug, should be considered as a therapeutic option in combination with trametinib, for patients with NRAS^Q61mut^ or other non-V600E BRAF mutant melanomas.

## INTRODUCTION

The paradigm of targeted therapy, the one-drug one-target disease approach, has issues including the development of resistance and, thus, there is a need for new therapeutics to target newly amplified or mutated proteins [1]. As cancer progresses by multiple pathways, targeting one pathway alone is usually insufficient. Consequently, combination targeted therapies have been advocated as a new approach to cancer treatment using either multi-target inhibitors or combinations of single-target agents. Thus, promiscuous inhibitors such as sorafenib and sunitinib are effective at disrupting multiple nodes in cell-signaling pathways. However, since these agents may have unacceptable side effects, dose titration is problematic [2]. The latest technologies used for new drug discovery are in part intended to circumvent these challenges, and while the number of new molecular entities introduced in 2014 increased, trends in drug development have been variable [3]. An alternative approach is to utilize available drugs and repurpose them for other indications.

Mebendazole (MBZ; methyl N-[6-(benzoyl)-1H-benzimidazol-2-yl] carbamate), an inexpensive chewable anthelmintic drug, has been widely used since the early 1970s [4] and is non-toxic even when administered in high doses [5]. MBZ acts at the colchicine-binding site of roundworm tubulin, and disrupts its polymerization [6, 7]. MBZ does not cause side effects characteristic of other anti-microtubule drugs, including taxanes and the vinca alkaloids [8].

The microtubule-disrupting properties of MBZ and other benzimidazole carbamates such as albendazole stimulated interest in these drugs as anti-cancer agents. MBZ inhibits mitotic spindles, induces G2/M arrest and apoptosis in human lung cancer cells, and suppresses their ability to form tumors in nude mice without host toxicity [9, 10]. However, the affinity of MBZ for human tubulin is much less than that of roundworm tubulin and it is unlikely that circulating MBZ levels would ever reach levels sufficient to block human tubulin *in vivo*. In addition to targeting tubulin, studies by our lab and others revealed that MBZ inhibits VEGFR-2, PDGFRA and PDGFRB at 3600 nM, 820 nM and 660 nM, respectively [11–13]. MBZ inhibits growth of melanoma cell lines *in vitro* and *in vivo*, accompanied by changes in tubulin polymerization, BCL2 phosphorylation and apoptosis [14]. While two clinical trials of MBZ for glioma are currently ongoing, two case reports for patients with either metastatic adrenocortical cancer or metastatic colon cancer highlighted evidence of clinical benefit. In the former case, some regression in metastatic lesions were observed, and the cancer remained stable on MBZ monotherapy, with the patient tolerating treatment without side effects until progression at 24 months [15]. In the latter case, near complete regression of metastatic lesions in lungs and lymph nodes and partial regression in the liver was observed [16]. A further five patients were treated with MBZ, including one experiencing a minor tumor response, in a reported personal communication [17].

Current cancer therapies have focused on targeting driver mutations, including oncogenic BRAF and NRAS, which are frequent in melanomas. BRAF^V600E^ and BRAF^V600K^ are found in 46% and 9% of melanomas, respectively. Additionally, 10% of melanomas previously classified as “BRAF^WT^” tumors actually harbor non-V600E/K mutations in BRAF. In fact, more than 30 mutations of the *BRAF* gene associated with human cancers have been identified [18], many of which may be sensitive to trametinib since these show deregulated stimulation of MEK1/2. Acquired resistance to the targeted therapeutics dabrafenib (GSK 2118436a; a BRAF^V600E/K^ inhibitor) and/or trametinib (GSK1120212; a MEK1/2 inhibitor) is associated with development of additional mutations, including those activating NRAS. Patients with melanomas harboring NRAS^mut^/BRAF^WT^ signatures (~21% of patients) have limited treatment options and are refractory to current targeted therapies.

In the current study, we report that the combination of MBZ and trametinib suppresses proliferation of patient-derived melanoma cell lines harboring NRAS^mut^/BRAF^WT^ as determined by gene sequencing, and significantly attenuates their growth in xenografts in immunocompromised mice. Reverse phase protein array (RPPA) based protein pathway activation mapping and immunoblot analyses revealed specific inhibition of the MAPK pathway and downstream regulation by MBZ or trametinib alone or in combination, within 10 minutes of drug treatment. At later time points, MBZ+trametinib induces markers of apoptosis, including proteolytic activation of caspase-3 and PARP cleavage, increased caspase activity as measured by fluorometric assays and increased levels of apoptotic sub-G1 cells. A reduction of cells in S phase was also observed in cells exposed to trametinib (by 24 h) or trametinib+MBZ (by 8 h), concurrent with an increase in G2 by 24h and in G1 by 48h. Thus, these results are consistent with the suppression of the MEK1/2 by trametinib and suppression of BRAF^WT^ by MBZ, leading to the combinatorial rapid shutoff of ERK activity, as well as downstream targets of ERK. MBZ is therefore a viable nontoxic option that can be used to increase the effectiveness of trametinib in NRAS^mut^/BRAF^WT^ melanoma.

## RESULTS

### *In silico* repurposing technology and *in vitro* kinase assays show that MBZ inhibits mutant and wild-type BRAF

**Figure 1:**
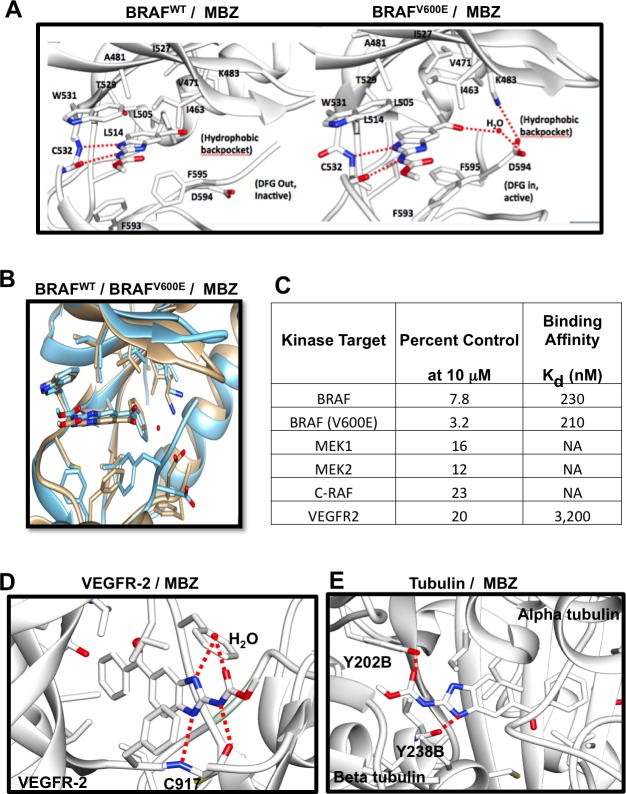
Structure of MBZ with BRAF^WT^ (left) or BRAF^V600E^ (right) are displayed showing residues critical for binding; hydrogen bonds are shown as dotted red lines **A**. Structure overlay of BRAF^WT^ (blue) and BRAF^V600E^ (tan) with MBZ **B**. MBZ inhibition of kinase activity and binding affinities for different targets **C**. Structure model of VEGFR-2 (PDB:2OH4; **D**.) and Tubulin (PDB:3N2G; **E**.) with MBZ. Critical binding site residues are displayed; hydrogen bonds shown as dotted red lines and water molecule shown as ball model.

Efforts to develop drugs targeting mutant BRAF led to FDA approval of vemurafenib in 2011 and dabrafenib in 2013. While these drugs either used alone or particularly when used in combination with MEK inhibitors such as trametinib or cobimetinib have been extremely successful at shrinking tumors, delaying disease progression and prolonging survival, resistance to them commonly develops at a median of 7-12 months typically through the selection of variants exhibiting mutations in other kinase pathway members, most notably NRAS. Our refined TMFS method [12] identified MBZ as a hit with a mode of inhibition that binds both wild type and V600E mutant BRAF (Figure 1A, 1B). In addition, other MAPK pathway proteins including CRAF and MEK were identified. Our *in-vitro* assays confirmed that BRAF and MEK were inhibited by MBZ in the nM range (Figure 1C), with MBZ inhibiting both BRAF^V600E^ and BRAF^WT^ with a K_d_ of 210 and 230 nM, respectively, in agreement with previous results with a kinase screen of MBZ, chosen for its ability to inhibit colon cancer growth [13].

Both sorafenib, a pan-kinase inhibitor that interacts with BRAF [18], and RAF265, a RAF/VEGFR dual kinase inhibitor [19], bind to the DFG-out (indicating the positions of the three key amino acids aspartate, phenylalanine, and glycine) inactive conformation of BRAF^WT^ and BRAF^V600E^ at the ATP binding site. In contrast, vemurafenib [20] and dabrafenib [21] bind to the DFG-in active conformation of the ATP binding site. These active conformation inhibitors are highly BRAF-selective compared to other kinases [20]. Our structure-based modeling shows that MBZ binds both inactive and active conformations of BRAF (Figure 1A, 1B). The BRAF structural model revealed that MBZ occupies the ATP-binding site and stabilizes both the active DFG-in and inactive DFG-out conformations. MBZ is surrounded by residues I463, V471, A481, K483, L505, L514, I527, T529, W531, C532, D594, and F595, and its binding is driven by hydrophobic and hydrogen bond interactions at the ATP site. An amide proton at the 2-position and a nitrogen atom at the N-1 position of the methyl N-(1H-benzimidazol-2-yl)carbamate moiety of MBZ form a significant hydrogen bond interaction with the backbone C = O and -NH of C532 in the kinase hinge regions of both the DFG-in and the DFG-out forms of BRAF^WT^ and BRAF^V600E^. The methyl group connected to amide moiety of methyl N-(1H-benzimidazol-2-yl)carbamate forms a hydrophobic interaction with the indole ring of W531, and is suitably placed, whereas larger hydrophobic replacements would create steric hindrance due to space constraints in the binding site between the indole side chain of W531 and G534. Two interactions between MBZ and BRAF^V600E^, but not BRAF^WT^ include: 1) an additional hydrophobic interaction between F593 and the benzimidazole moiety, and 2) a water-mediated hydrogen bond interaction between D594 and a keto group of the methyl N-(1H-benzimidazol-2-yl)carbamate moiety. These differences explain the slightly higher K_d_ value observed for BRAF^WT^. MBZ does not interact with the BRAF lipophilic back pocket, unlike other BRAF inhibitors, lowering its affinity for C-RAF.

TMFS analysis also reveals that MBZ interacts with VEGFR2 (Figure 1D), consistent with its structural similarity to the benzimidazole-urea VEGFR2 inhibitor (PDB:2OH). However, MBZ showed more potency towards BRAF (Figure 1C) than to VEGFR2 [12], probably due to the absence of its interaction with residues lining the ATP site back pocket, which is more important for VEGFR2 than for BRAF^V600E^. In addition, the F918 phenyl ring of VEGFR2 restricts the diversified and non-planar conformation of MBZ, compared to the W531 indole ring of BRAF. Consistent with previous studies [14], MBZ also interacts with tubulin (Figure 1E).

### The combination of MBZ and trametinib is cytotoxic to NRAS- and BRAF-mutant melanoma cells

**Figure 2:**
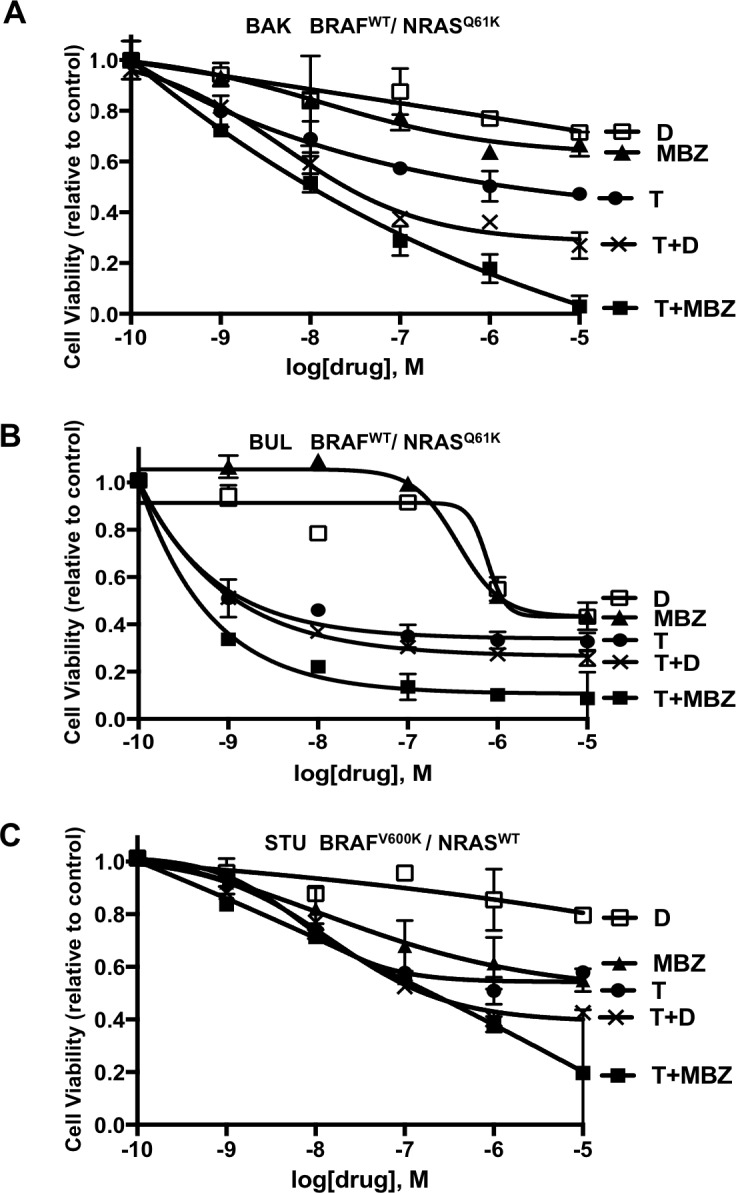
Decreased viability of melanoma cells exposed to MBZ, dabrafenib (D), trametinib (T), or combinations of T+D or MBZ+trametinib **A**. BAK (BRAF^WT^/NRAS^Q61K^), **B**. BUL (BRAF^WT^/NRAS^Q61K^) and **C**. STU (BRAF^V600K^/NRAS^WT^) melanoma cells were exposed to the indicated concentrations of MBZ, D, T, T+D or MBZ+trametinib (T+MBZ) for 72 h, and subjected to XTT cell viability assays, as described in Materials and Methods. Error bars represent mean ± SD for triplicates. Significant growth inhibition was observed at ≥ 1 nM for T or T+MBZ, ≥ 10 nM for MBZ alone, and ≥ 1 µM for D (2-way ANOVA). The results shown are based on a single experiment in triplicate, and repeated in three independent experiments with essentially the same results.

Based on the ability of MBZ to target both mutant and wild-type BRAF, two patient-derived melanoma cell lines (BAK and BUL) harboring the same BRAF^WT^/NRAS^Q61K^ mutation profile and another melanoma cell line (STU) with a BRAF^V600K^/NRAS^WT^ mutation signature were exposed for 72 h to increasing concentrations of dabrafenib (D), trametinib (T), MBZ, or combinations of T+D or T+MBZ. XTT cell viability assays revealed that, while all three cell lines exhibited resistance to dabrafenib except at the highest doses tested, MBZ showed weak cytotoxic activity as a single agent, but synergized strongly with trametinib in both BAK and BUL cells, but was either antagonistic (at low concentrations) or additive (at high concentrations) in STU cells. T and D were also synergistic in BAK and BUL, but not STU, although maximum inhibition was greater in T+MBZ - treated cells (Figure 2; Supplementary Table 2, Supplementary Figure 1). Consequently, the MBZ+trametinib combination may represent a potential therapy in NRAS mutant melanoma cells.

**Figure 3:**
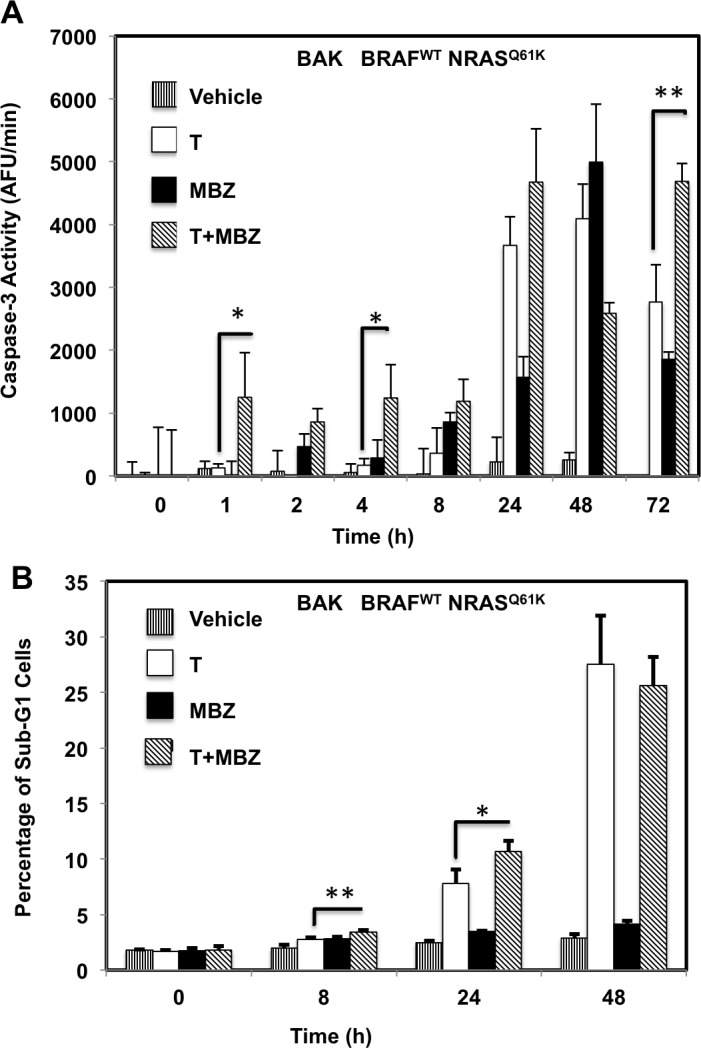

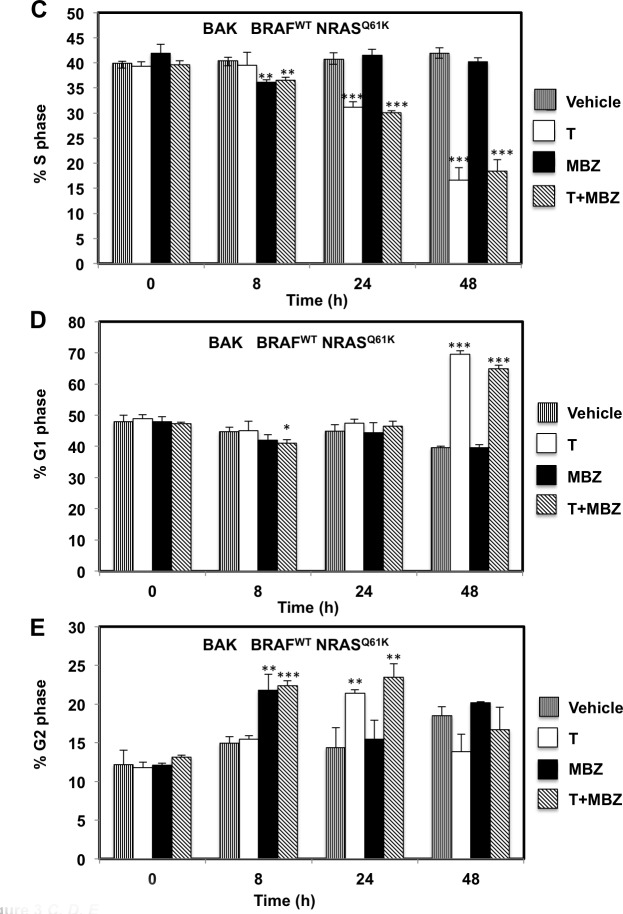

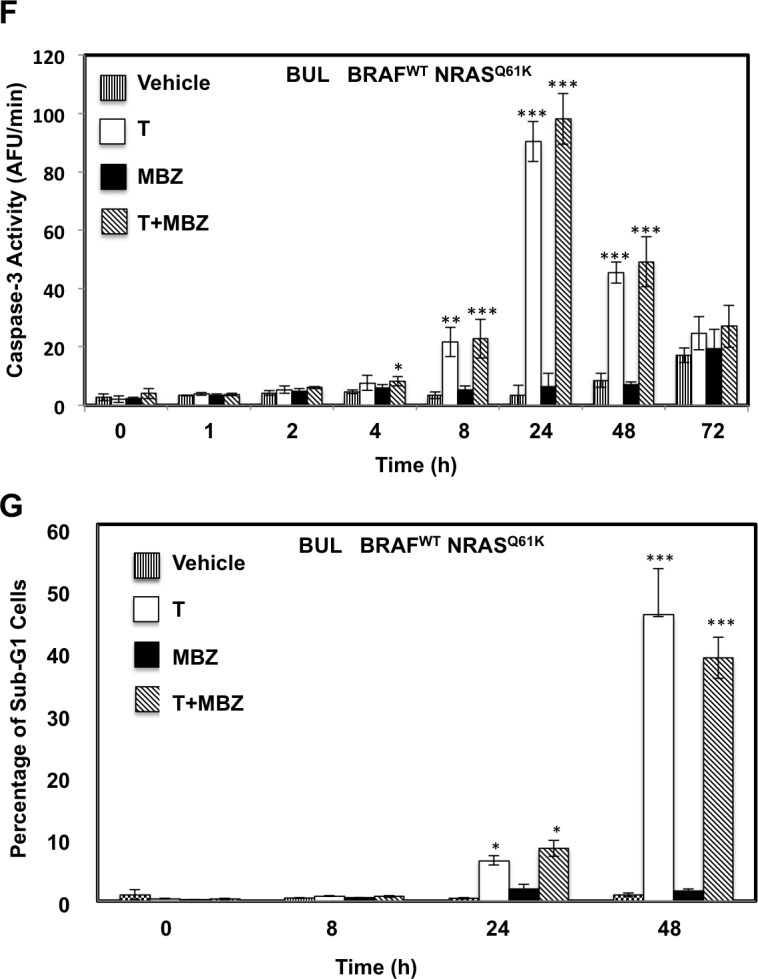

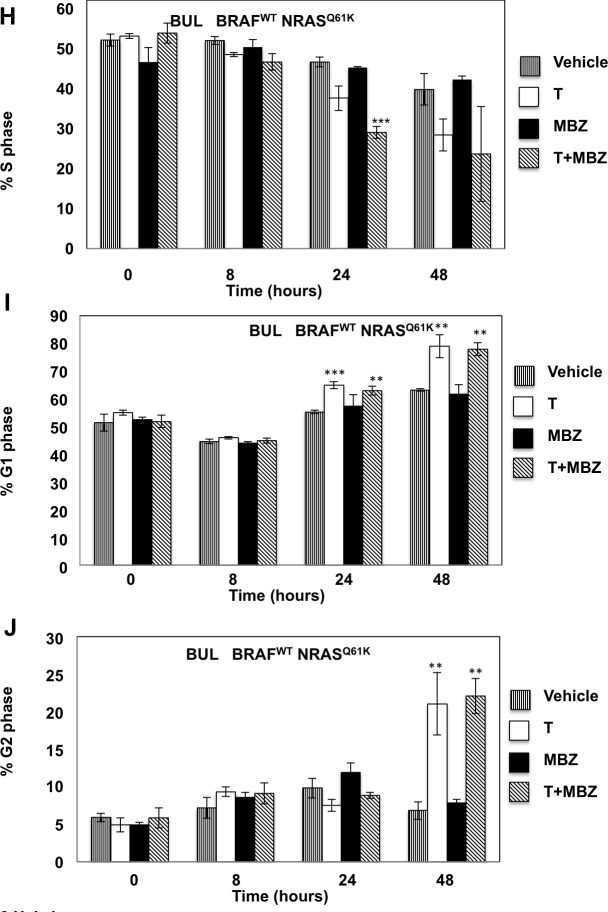
MBZ+trametinib induces apoptosis and decreases the percentage of melanoma cells in S phase of the cell cycle BAK **A**.-**E**. or BUL **F**.- **J**. melanoma cells were exposed to 100 nM of MBZ, trametinib, or a combination of the two (T+MBZ); cytosolic extracts were derived at indicated times and subjected to fluorometric analysis using DEVD-AMC as a substrate (A, F), or cells were fixed in EtOH, stained with PI, and subjected to FACS analysis to determine the number of cells in sub-G1 (B, G), S phase (C, H), G1 phase (D, I), or G2 phase of the cell cycle (E, J). Results are the means ± S.D. of three replicates of a representative experiment. Statistical analysis of T versus T+MBZ groups (A, B) and between vehicle and treatment groups (C-J); 1, 2, or 3 asterisks (*) represent *p* < 0.05, *p* < 0.001, and *p* < 0.0001, respectively.

To determine whether the reduced cell numbers were due to inhibition of proliferation or increased cell death, apoptosis and cell cycle assays were performed in BAK and BUL melanoma cells. Caspase-3 activity (Figure 3A, 3F), as well as a sub-G1 population (Figure 3B, 3G) was induced earlier, and to a greater extent in cells exposed to T+MBZ than to either drug alone, indicating that this combination rapidly and robustly induces apoptosis. In both BAK and BUL cells, trametinib and/or the combination of T+MBZ also decreased the percentage of cells in S phase of the cell cycle at all time points, with concomitant increases in G2 and G1 phases of the cell cycle by 8 h or 24 h, respectively (Figure 3C-3E, 3H-3J).

### The combination of MBZ and trametinib reduces tumor growth in xenografts

To determine if MBZ and trametinib are effective against BRAF^WT^ /NRAS^Q61K^ melanoma *in vivo*, BAK cells were xenografted into nude mice, and treated with MBZ, trametinib, or their combination. Two different doses of trametinib were administered to different groups of mice daily by gavage (0.1 mg/kg LDT, or 3 mg/kg HDT). A third group of mice was treated with a dose of MBZ similar to that used for helminthic infections (40 mg/kg on alternate days), while a fourth and fifth group of mice received a combination of MBZ and either LDT or HDT. These trametinib doses bracket those prescribed for patients (from ¼- to 7-fold), and our MBZ doses are much less than those safely used in patients based on dose per body surface area (BSA; Materials and Methods), and are similar to those in previous preclinical studies [22, 23]. The vehicle served as a control for the sixth group of mice.

**Figure 4:**
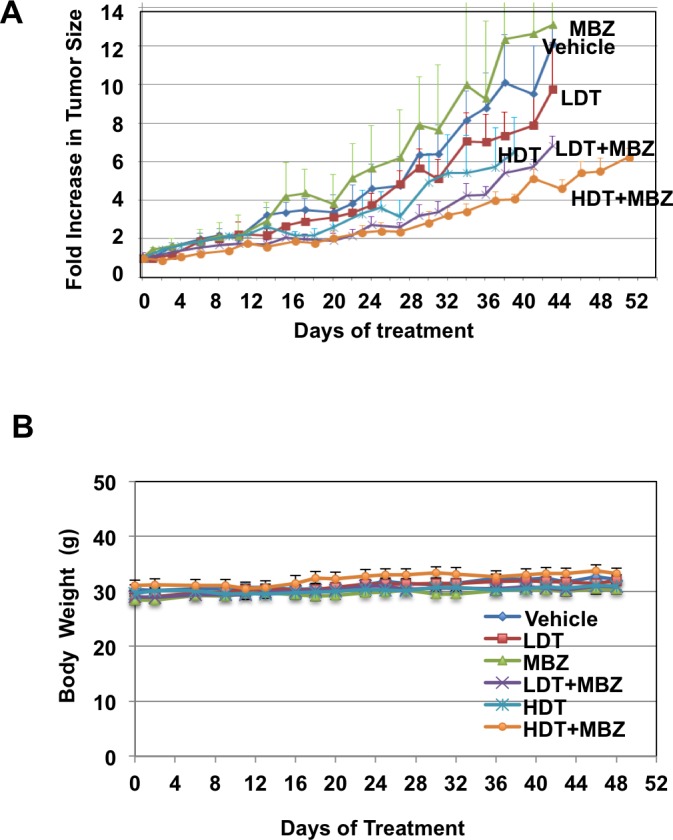

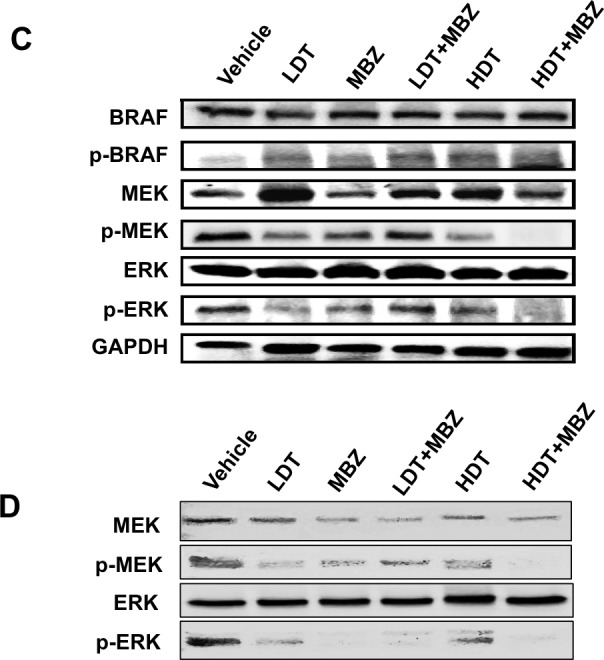
MBZ+trametinib significantly inhibit tumor growth and phosphorylation of MEK and ERK ***in vivo***. Athymic mice were injected with 3 × 10^6^ BAK melanoma cells, and tumors were allowed to grow to a volume of 100 mm^3^. Animals were then gavaged with vehicle emulsion control, 40 mg/kg/qad MBZ, low dose trametinib (LDT; 0.1 mg/kg/qd), high dose trametinib (HDT; 3 mg/kg/qd) or a combination of MBZ and LDT or HDT. **A**. Tumor widths and lengths were measured and volumes calculated as *w*^2^ *x l/2*, where width is defined as the smaller of the tumor dimensions. Time 0 is the tumor volume on the first day of treatment; tumor sizes were normalized to their size at time 0 of drug treatment. **B**. Mice were weighed every other day and body weights (g) plotted over time. Data from four experiments was combined for statistical analysis, to compare every mouse from each treatment group. The total mice for all experiments included vehicle control (*n* = 13), LDT (*n* = 12), MBZ (*n* = 12), HDT (*n* = 12), LDT+MBZ (*n* = 12), and HDT+MBZ (*n* = 15). The results are shown as the mean (±SD) of tumor volume in each group. **C**.*,* **D**. ERK and MEK phosphorylation is suppressed in large (C) or small (D) tumor xenografts from mice treated with T+MBZ. Tumor extracts were derived from xenografts, then subjected to immunoblot analysis using antibodies specific for total BRAF, phospho-BRAF, ERK1/2, phospho-ERK1/2, total MEK1/2, phospho-MEK1/2, or GAPDH as a loading control.

While trametinib as a single agent did not show any significant tumor-suppressive effects (HDT *vs*. control *p* = 0.26; LDT *vs*. control *p* = 0.65, Supplementary Table 1), tumor growth was significantly inhibited in mice treated with MBZ in combination with either high (HDT+MBZ *vs*. vehicle *p* = .038) or low (LDT+MBZ *vs*. vehicle *p* = .066) trametinib doses, although not quite to a significant level in the latter case, without loss in weight or any other obvious adverse effects (Figure 4A, 4B). Remarkably, the HDT+MBZ combination group remained alive long after the other arms had been euthanized due to the size of the NRAS^Q61K^ melanoma xenografts at 42 days. Tumors from xenografts collected at the termination of the experiment were then subjected to immunoblot analysis to determine the protein levels and phosphorylation status of components of the MAPK pathway *in vivo*. Whereas MBZ and trametinib alone each demonstrate the ability to suppress MEK and ERK phosphorylation, only the combination of HDT+MBZ completely abrogated both MEK1/2 and ERK1/2 phosphorylation (Figure 4C, 4D).

### MBZ and trametinib target the MEK/ERK pathway

**Figure 5:**
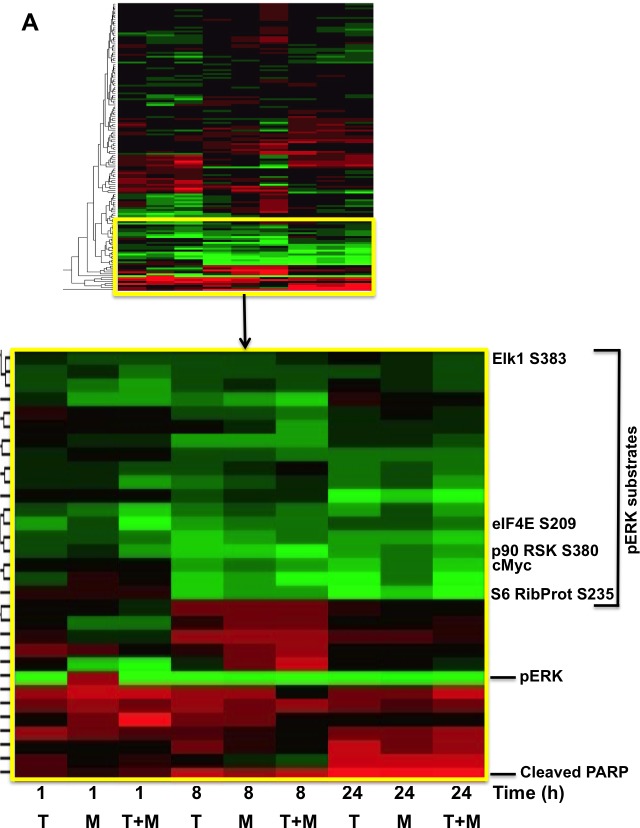

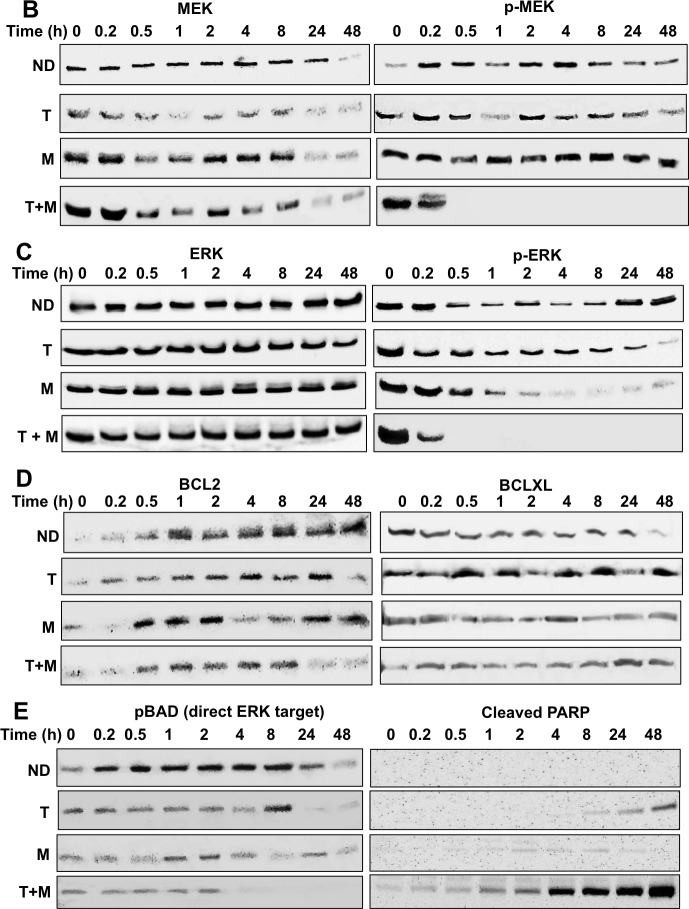

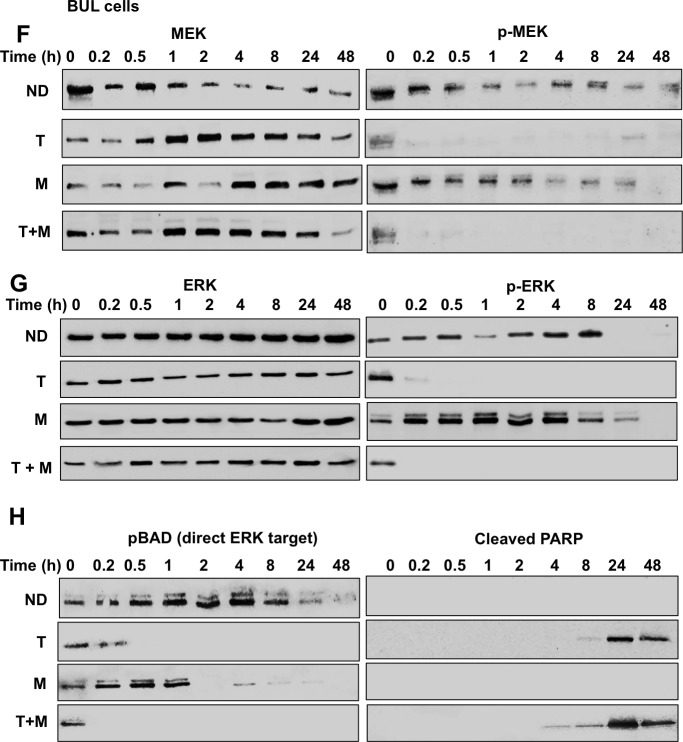
RPPA and immunoblot analyses reveal suppression of the MAPK pathway, including ERK and its downstream substrates in melanoma cells exposed to MBZ+trametinib **A**. BAK cells were exposed to 100 nM of MBZ, trametinib, or a combination of the two. Cell extracts derived at indicated times were subjected to RPPA analysis, and unsupervised hierarchal clustering was used to generate a heat map as described in Materials and Methods. **B**.-**E**. Immunoblot analyses show marked attenuation of pMEK, pERK, as well as pBAD, coincident with increased levels of cleaved PARP, in melanoma cells exposed to MBZ+trametinib. BAK cells were treated with 100 nM of MBZ, trametinib, or a combination of the two for the indicated times; cell extracts derived and subjected to immunoblot analysis with antibodies specific for total MEK1/2, phospho MEK 1/2 (B), total ERK1/2, phospho-ERK1/2 (C), BCL2, BCLXL (D), phospho-BAD and cleaved PARP (E). All immunoblots were then reprobed with GAPDH as loading control (Supplementary Figures 3-4).

Given the potential changes in tumor cell signaling and survival over the long time course of the xenografts, we examined potential mechanisms by which MBZ+trametinib exerts its cytotoxic effects using cultured BAK NRAS^Q61K^ melanoma cells. Cells were exposed to MBZ (10 nM or 100 nM), trametinib (10 nM or 100 nM), or a combination of the two for 1, 8, or 24 h, after which cell extracts were subjected to reverse-phase protein array (RPPA) analysis. Unsupervised hierarchal clustering of rows revealed that the phosphorylation of a number of proteins associated with the MEK/ERK pathway was down-regulated by MBZ, trametinib, or their combination, although the response to MBZ+trametinib (T+M) was more rapid and robust (Figure 5A). Thus, phosphorylation of ERK and its downstream targets involved in translation, including p90RSK, ribosomal protein S6, and eIF4E were all concomitantly inhibited within 1 h of drug exposure, and remained hypophosphorylated for 24 h in the T+M groups; hypophosphorylated ELK1 S383 (a known ERK kinase substrate) was also associated with this cluster (Figure 5A; Supplementary Figure 2A*).* Levels of LC3B and Beclin-1, key regulatory proteins that control autophagy, and known ERK pathway substrates, were also reduced in this cluster [24, 25]. Proteins characteristic of apoptosis were elevated with time, including the proteolytically activated form of caspase-3 and cleaved PARP, while total levels of the cell cycle progression protein cMYC were reduced (Figure 5A; Supplementary Figure 2B), consistent with the effects of MBZ and trametinib on these two processes (Figure 3).

The potential pathway for early suppression of phospho-ERK and its targets, leading to apoptosis and cell cycle suppression was next examined by immunoblot analysis. Remarkably, whereas the pMEK1/2 S217/221 activating phosphorylation was not inhibited by trametinib or MBZ alone, their combination completely abolished detectable MEK phosphorylation within 30 min (Figure 5B). While levels of total ERK1/2 remained constant throughout the time course for all treatment groups, the activating phosphorylation of ERK1/2 (T202/Y204) was diminished by MBZ or trametinib alone. However, the combination of MBZ+trametinib completely abrogated ERK phosphorylation, such that phospho-ERK was undetectable by 30 min of treatment (Figure 5C). Consistent with the regulation of BCL2 levels by MEK/ERK [26], BCL2, but not BCLXL levels, were slightly reduced by MBZ+trametinib by 24 h (Figure 5D). Further, cells treated with the MBZ+trametinib combination exhibited marked suppression of the inactivating phosphorylation of the pro-apoptotic ERK substrate BAD S11 [27], coincident with a time-dependent increase in cleaved PARP (Figure 5E), which is consistent with results observed by RPPA analysis (Figure 5A; Supplementary Figure 2B).

**Figure 6:**
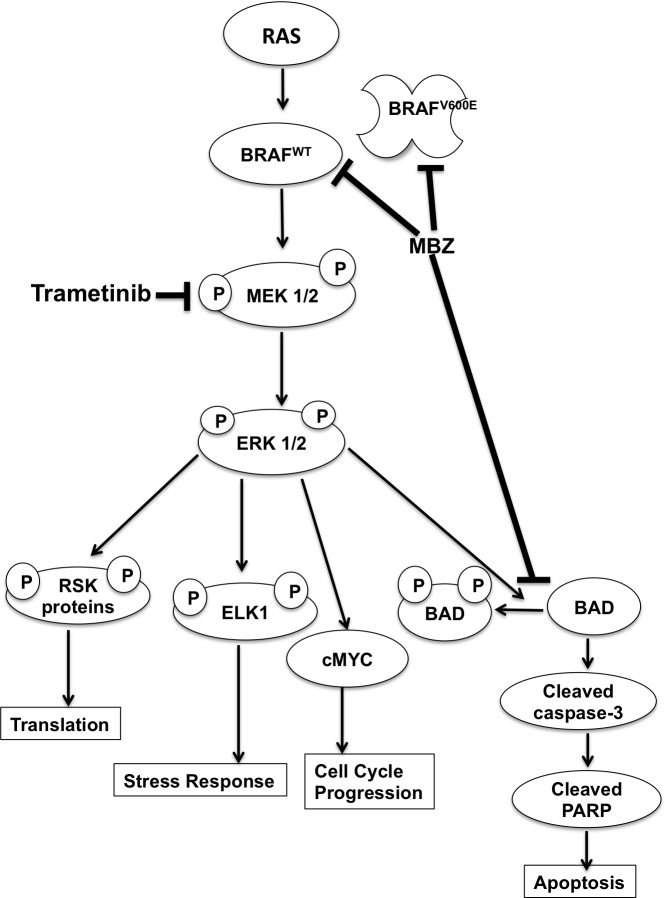
Model of MBZ+trametinib mechanism of action in melanoma cells

The immunoblot experiments were repeated with an additional melanoma cell line BUL, which harbors the same NRAS^Q61K^ mutation. Similar to BAK, BUL cells also exhibit a marked attenuation of ERK and MEK phosphorylation within 30 min of T+MBZ exposure, coincident with a loss of BAD phosphorylation and increased cleavage of PARP (Figure 5F-5H), demonstrating that both mutant NRAS cells respond strongly to the combination of these two drugs. Taken together, a proposed model for suppression of melanoma growth by MBZ+trametinib is shown in Figure 6.

## DISCUSSION

Our previous results [12] showed that MBZ interacts with VEGFR *in silico*, which was subsequently corroborated by other investigators who showed that MBZ interacts with additional kinases *in vitro*, including BCR-ABL and BRAF [13, 16]. In the current study, we expanded these findings using TMFS to show the nature of the interactions between MBZ and VEGFR, and with BRAF^WT^ or BRAF^V600E^. We also now report the novel finding that MBZ binds both the active and inactive forms of these BRAF proteins. The current study focuses primarily on NRAS^mut^/BRAF^WT^ melanoma cells, which account for about 21% of all melanoma cases [28–31]. These have been particularly recalcitrant to treatment, with overall survival times that are shorter than those of patients with melanoma harboring BRAF mutations, and do not respond to BRAFV600 inhibitors such as vemurafinib and dabrafenib. In fact these inhibitors actually enhance growth of NRAS^mut^/BRAF^WT^ tumors by paradoxically further activating the MAPK pathway through induced conformational changes in wild type RAF isoforms, inducing dimerization, membrane localization, and activation by RAS [32]. The advantages of MBZ are that 1) it interacts with both the active and inactive forms of BRAF, 2) it binds wild type or mutant BRAF with almost equal affinities, and 3) it has very low affinity for CRAF, and therefore would not be expected to stimulate tumor growth.

Consistent with our TMFS and kinase assays, MBZ is toxic to patient-derived melanoma cells harboring either WT or mutant BRAF in the presence of trametinib. Further, MBZ+trametinib strongly suppressed the growth of BRAF^WT^/NRAS^Q61K^ melanoma xenografts, and dramatically inhibited ERK1/2 phosphorylation within 10 min. This also blocked phosphorylation of its downstream targets resulting in suppression of proliferation, inhibition of autophagy, and induction of apoptosis; at least in part *via* suppression of ERK-mediated phosphorylation of BAD, ELK1, decreased expression of Beclin and LC3B, and decreased BCL2 levels. In light of the relative non-toxicity of MBZ, we propose that the MBZ-trametinib combination is a compelling candidate as a therapeutic for patients with metastatic NRAS mutant melanoma.

Previous studies showed the efficacy of MBZ in melanoma cell culture and preclinical mouse models [14, 22, 23]. However, in our mouse xenograft model, MBZ did not work as a monotherapy, but did strongly enhance the effects of trametinib (Figure 4). This might be due to the difference in mutation profiles in our study in which BAK NRAS^Q61K^/BRAF^WT^ primary human melanoma cells were used rather than the long-term culture M14 melanoma cells harboring a NRAS^WT^/BRAF^V600E^ mutation profile in the previous study. Consistent with the previous study showing BCL2 down-regulation in cells treated with MBZ, our results likewise demonstrate that MBZ+trametinib reduces BCL2 levels, as well as BAD S112 phosphorylation, both of which can be explained by the inhibition of MEK/ERK by MBZ+trametinib [26, 27], although some contribution by a lower affinity MBZ-tubulin interaction cannot be ruled out. Additionally, we observe inhibition of phospho ELK1 S383 at all time points and treatments, a mechanism previously shown to mediate sorafenib-induced endometrial carcinoma apoptosis by lowering MCL1 levels [33] (Figure 5A). MBZ-induced increase in G2 levels was noted (Figure 3E, 3J), consistent with previous findings in human lung cancer cells, [9, 10]. Other differences in the response to MBZ observed previously may be due to the differences in melanoma cell lines, although it should be pointed out that the BAK cells used in the current study harbor a difficult to treat mutation profile.

An important question is whether MBZ and trametinib can reach sufficient concentrations in patients to exert the anti-tumor effects observed in our current cell culture and mouse xenograft studies. One challenge is the relatively poor absorption of MBZ through the gut, which has been unnecessary for the treatment of nematode and cestode parasites resident in the human digestive tract, where it is believed to function by binding tubulin with an apparent binding affinity of 19 nM, but has a much lower affinity for human tubulin (µM range; [6]. However, our results suggest that a major target for MBZ is in fact BRAF, with a higher affinity (Figure 1C) that might be obtained in patients. Oral MBZ can reach peak serum concentrations similar to those used in our current study. For example, in patients treated with chronic MBZ for hydatid disease, a dose of 10 mg/kg/day resulted in a mean peak plasma level of 470 nM, with some variability between patients (0.34-1.69 μM) [34], matching half the dose (or 6X the BSA-adjusted dose; Materials and Methods) administered to the mice in this study (40 mg/kg/qad; Figure 4), and a plasma concentration equivalent to an IC80 in our cultured NRAS cells (Figure 2A). We are currently testing different formulations of the drug to achieve higher bioavailability and plasma concentrations. MBZ significantly enhanced efficacy of trametinib at 0.1 mg/kg/day (LDT) or 3 mg/kg/day (HDT). Our trametinib doses/[mouse BSA] bracket those prescribed for patients (from ¼- to 7-fold), and our MBZ doses are similar to those used in patients (Materials and Methods) and have been used in previous animal studies [22]. In any case, the trametinib dose-dependent reduction in growth of the MBZ-trametinib treatment groups is promising considering the difficulty treating NRAS tumors, and may work in concert with other drugs. We have collaborated with colleagues at Johns Hopkins who have identified a formulation of MBZ with significantly greater oral absorption. We have also developed an investigator-initiated Phase IA/B clinical trial using this new formulation of MBZ in combination with a MEK inhibitor for patients with NRAS mutant melanoma that is currently under review at a major pharmaceutical company. Based on the encouraging synergy, and likely tolerability of this combination, we are hopeful that the trial will be approved enabling us to formally test this combination in patients.

## MATERIALS AND METHODS

### Proteochemometric methods

A novel rapid computational Proteochemometric method called “Train, Match, Fit, Streamline” (TMFS) was used to map new drug-target interaction space and predict new uses as described [12]. The TMFS method combines shape, topology and chemical signatures, including docking score and functional contact points of the ligand, to predict potential drug-target interactions with remarkable accuracy.

### Establishment and characterization of primary human melanoma cell lines

Human melanoma cell lines were established from fresh metastatic tumor tissues of consenting patients. Tumors were analyzed for mutations in *CKIT, BRAF*, and *NRAS* by next generation sequencing. Single cell suspensions were prepared from freshly resected tumor tissue specimens by mechanical mincing; no enzymatic dissociation was used. Viable tumor cells were cultured in Iscove's Modified Dulbecco's Medium (IMDM) supplemented with 10% fetal bovine serum (FBS) and antibiotics. After overnight incubation at 37˚C in 5% CO_2_, floating debris was discarded and fresh complete medium was added. Cultures were fed 2-3 times per week, replacing half of the spent medium. Melanoma cell lines were split when near confluence and sub-cultured at 4×10^4^ viable cells per cm^2^ surface area in flasks. Cultures were shown to be free of mycoplasma contamination using the MycoProbe™ mycoplasma detection kit (R&D Systems, Minneapolis, MN, USA). To ensure that cultured cell lines were melanoma cells, each cell line was stained and analyzed by flow cytometry for melanoma-specific antigens MART-1, gp100, TRP75, or melanoma-associated chondroitin sulfate proteoglycan. All cell lines were early passages of less than 20.

### Cell culture

Patient-derived melanoma cell lines (BAK and BUL) with the same BRAF^WT^/NRAS^Q61K^ mutation signature, as well as a melanoma cell line (STU) harboring a BRAF^V600K^/NRAS^WT^ mutation were cultured in IMDM with 10% FBS and 1% penicillin/streptomycin in a 5% CO_2_ incubator at 37°C. Cell growth was monitored daily and expanded to obtain sufficient cell numbers for subsequent experiments. Mutation signatures of cell lines were confirmed by PCR and sequencing.

### Drug toxicity assays

MBZ, dabrafenib, and trametinib were purchased from Sigma-Aldrich and ActiveBiochem, respectively. 5 × 10^3^ viable cells per well were plated in 96-well dishes and allowed to recover for 12 h prior to drug treatment. Cells in triplicate wells were treated for up to 72 h (based on initial time course experiments showing maximal effects at that time point) with different concentrations of trametinib, dabrafenib, or MBZ alone, or a combination of MBZ and trametinib. Negative controls were exposed to vehicle DMSO in the same volumes. Cell viability was assessed by an XTT assay, according to a manufacturer's specifications (Biotium Inc). Reduced XTT was measured by absorbance at 490 nm on a PerkinElmer Victor3 plate reader. Cells exposed to detergent served as a positive control.

### Cell cycle analysis

Cells were collected, fixed in ethanol, stained with propidium iodide (PI) to determine DNA content, and analyzed by flow cytometry (FACStar PLus; BD BioSciences, San Jose, CA).

### Mouse xenografting

All animal experiments were performed in accordance with the guidelines and approval of Georgetown University Animal Care and Use Committee. Athymic 6-week old male mice (Taconic) were acclimated to the Division of Comparative Medicine at Georgetown University a week prior to xenografting. 3×10^6^ melanoma cells were resuspended in Matrigel and injected subcutaneously into the hind flanks of athymic mice using a 22-gauge needle. Tumor growth was measured with calipers, and drug treatment started when tumor volumes reached 100 mm^3^, after which mice were monitored daily for drug efficacy, as well for adverse effects, including weight and behavior. Drugs were administered by oral gavage. Each testing group contained three to five mice in each of four experiments. Each tumor from each treatment group was measured on indicated days, and all tumor sizes were then normalized to their size at day 0 of drug treatment. All data from all four experiments was then combined for statistical analysis, to compare every mouse from each treatment group. The total mice for all experiments included vehicle control (*n* = 13), low-dose T (*n* = 12), MBZ (*n* = 12), high-dose T (*n* = 12), low-dose trametinib +MBZ (*n* = 12), and high-dose trametinib +MBZ (*n* = 15). The results were expressed as the mean (±SD) of tumor volume in each group. After five weeks, mice were euthanized; tumors extracts were derived for immunoblot analysis.

### Dosing

Trametinib doses were calculated relative to doses prescribed for patients, based on weight and body surface area for mouse and human, using the surface area to weight ratios (m^2^/kg) described for mouse (.02 kg/ 0.0066 m^2^ = 3.0) and human (60 kg/1.6 m^2^ = 37) [35]. This yields a similar constant to that calculated by Mosteller for humans: BSA (m^2^) = [SQRT (H (cm) *x* W (kg)]*60 [36]. Our mouse “low dose” trametinib (0.1 mg/kg), adjusting for surface area = 3 /37* (0.1 mg/kg) = 0.008 mg/kg, is therefore equivalent to a human dose of 0.008 mg/kg * 60 kg (average body mass globally) = 0.48 mg/person/day. For high dose trametinib, this is equivalent to 14.4 mg/person/day. By comparison, the dose for patients is 2 mg PO qDay. For MBZ, our dose of 40 mg/kg, adjusted for surface area constants 3/37 (mouse/human), is equivalent to 3/37* (40 mg/kg) * 60 kg/person = 195 mg. By comparison, patient doses range from 100 mg one time (pinworms) to 200 mg/kg per day for 12 weeks (hyatid disease in children) including doses up to 6 g per day [5]. In summary, our trametinib doses/[mouse BSA] bracket those prescribed for patients (from ¼- to 7-fold), and our MBZ doses are much lower than those that have been safely used in patients [5, 37], and similar to those used in other preclinical studies [22, 23].

### Immunoblot analysis

SDS-PAGE and transfer of separated proteins to nitrocellulose membranes were performed according to standard procedures. Membranes were stained with Ponceau S (0.1%) to verify equal loading and transfer of proteins, and then incubated with antibodies specific for pERK1/2 T202/Y204, pMEK1/2 S217/221, pBAD S-112, total BAD, total ERK1/2, total MEK1/2, BCL2 (Santa Cruz Biotech), BCLXL (Santa Cruz Biotech), cleaved PARP (Cell Signaling), or GAPDH (Abcam; loading control). Immune complexes were detected by incubation with appropriate horseradish peroxidase-conjugated antibodies to mouse or rabbit IgG (1:3000) and enhanced chemiluminescence (Pierce, Rockford, IL).

### Fluorometric caspase-3 activity

Cytosolic extracts, derived from pooled floating and attached cells, were subjected to fluorometric caspase-3 activity assays using fluorescent tetrapeptide substrate specific for caspases-3 (Ac-DEVD-aminomethylcoumarin (AMC, Enzo Life Sciences, Ann Arbor, MI) as previously described [38]. Free AMC, generated as a result of cleavage of the aspartate-AMC bond, was monitored over 30 min with a Wallac Victor^3^ fluorometer (Perkin-Elmer, Waltham, MA) at excitation and emission wavelengths of 360 and 460 nm, respectively. The emission from each sample was plotted against time, and linear regression analysis of the initial velocity (slope) for each curve yielded the activity.

### Reverse-phase protein arrays

Cell lysates were analyzed by reverse-phase protein array (RPPA) [39]. Samples were diluted to 0.5 mg/mL and dilutions printed on slides in triplicate. Slides were immunostained with 137 different antibodies specific for total proteins, or phosphorylated or cleaved products. Analytes measured were chosen based on their ‘actionability” (*e.g*. were known drug targets for FDA-approved drugs, drugs in clinical trials, or targets of other commercially-available compounds), as well as for and their known involvement in tumorigenesis and cancer biology and components in key signaling pathways that control cell growth, motility, inflammation, autophagy, survival, differentiation and apoptosis. All antibodies have been pre-validated for specificity by immunoblot analysis. Intensity values were normalized to that of total protein for each sample stained with Sypro Ruby (Invitrogen). Unsupervised cluster analysis http://www.hiv.lanl.gov/content/sequence/HEATMAP/heatmap.html was performed for all proteins in the RPPA using the standard bootstrap method.

### Statistical analysis

The results shown are based on a single experiment in triplicate, and repeated in three independent experiments with essentially the same results. Data from triplicates of treatment groups were compared using Student's t-test or 2-way ANOVA (multiple comparisons) for significance, and *p* values of < 0.05 were considered statistically significant. For tumor sizes, the rate-based T/C (tumor/control) test of significance was used as described, using the author's template [40]. The results are representative of 3 independent experiments with reproducible results. For determining synergism, the combination index (*τ*) was calculated from single dose-response curves and combination experiments as *τ* = *x*_A_/X_A_+*x*_B_/X_B_, in which, for a given cytotoxic effect, *x*_A_ and *x*_B_ are the concentrations of drugs *A* and *B* in the combination, and X_A_ and X_B_ are the concentrations of drugs *A* and *B* that achieve the same cytotoxic effect when given alone [41]. A *τ* value of 1 indicates additivity, *τ* less than 1 indicates synergy, and *τ* greater than 1 indicates antagonism.

## SUPPLEMENTARY MATERIALS FIGURES AND TABLES


