# Supplementary material for: The repurposed anthelmintic mebendazole in combination with trametinib suppresses refractory NRASQ61K melanoma
Source: Oncotarget. 2017 Feb 2;8(8):12576–95. doi: 10.18632/oncotarget.14990 (PMC5355037; doi:10.18632/oncotarget.14990)
Supplement: Supplementary file 1 [file oncotarget-08-12576-s001.pdf]

The repurposed anthelmintic mebendazole in combination with trametinib suppresses refractory NRAS^{Q61K} melanoma

Supplementary Material

A BAK cells

T dose-response:
y1 <- 77.524-3.640*log(Concentration)

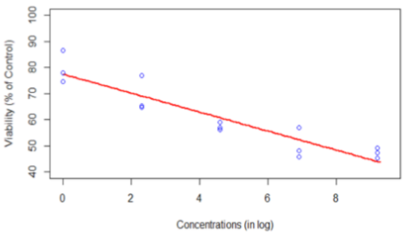

MBZ dose-response:
y1 <- 91.69-3.155*log(concentration)

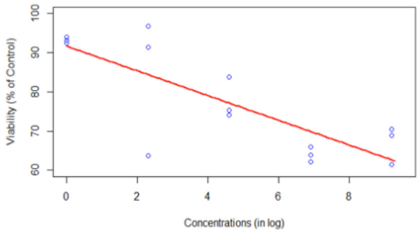

D dose-response:
y1 <- 93.492-2.298*log(concentration)

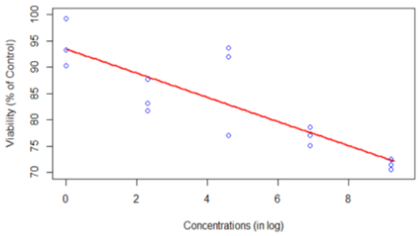

Combination Index of **T and MBZ** at dose 1, 10, 100, 1000, 10000 nM are 0.2553855, 0.0116237, 0.00035, 0.000168, 0.000018, respectively.
All combinations are synergistic.

Combination Index of **T and D** at dose 1, 10, 100, 1000, 10000 nM are 7.841233194, 0.226276739, 0.002946497, 0.019861052, 0.020159478, respectively.
All combinations are synergistic *except* at the combination of T=1 and D=1 where it is antagonistic.

B BUL cells

T dose-response:
y1 <- 49.021-2.122*log(Concentration

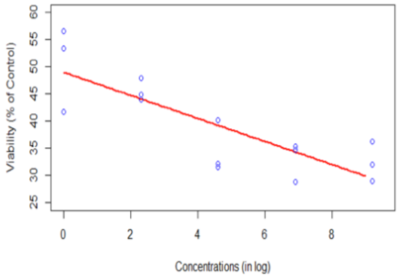

MBZ dose-response:
y1 <- 100.338-35.118*log(log(concentration)-3.5)

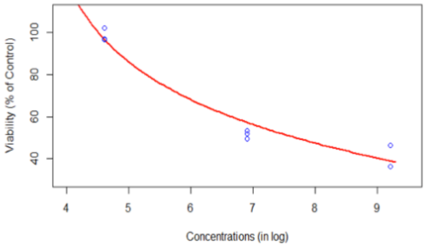

D dose-response:
y1 <- 96.896-5.431*log(concentration)

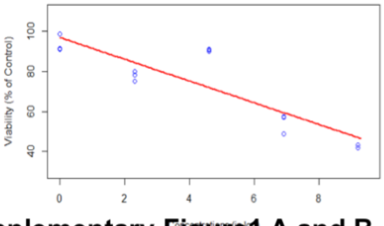

Combination Index **of T and MBZ** at dose 1, 10, 100, 1000, 10000 nM are 7.530693e-04, 7.310080e-05, 5.691205e-05, 7.689975e-05, 1.405457e-02, respectively. **All combinations are synergistic.**

Combination Index of **T and D** at dose 1, 10, 100, 1000, 10000 nM are 2.29282989, 0.03043452, 0.02175783, 0.04063049, 0.29565493, respectively.
All combinations are synergy except at the combination of T=1 and D=1 is antagonistic.

Supplementary Figure1 A and B

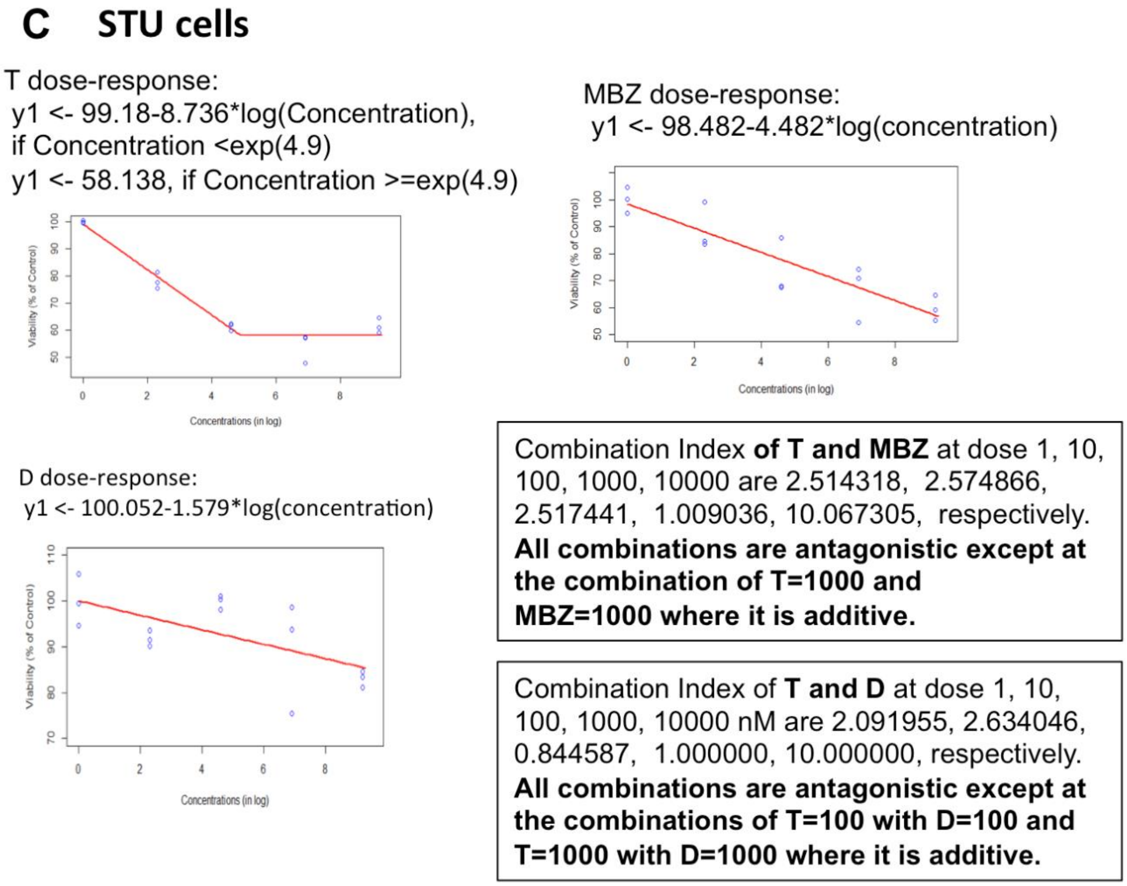

Supplementary Figure 1: Synergy analysis graphs for BAK (A), BUL (B) and STU (C) showing data summarized in Supplementary Table 2.

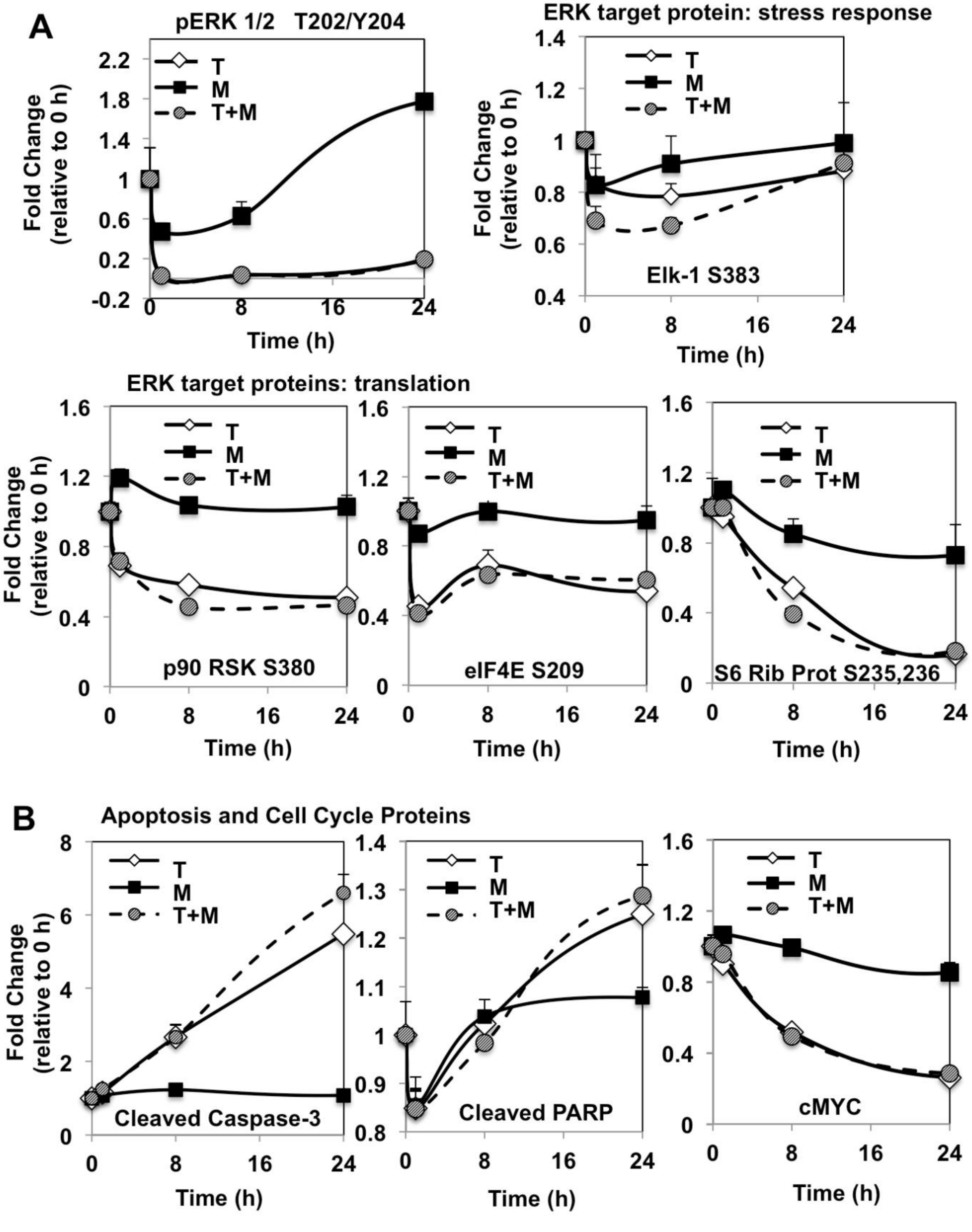

Supplementary Figure 2 A, B

Supplementary Figure 2: RPPA analyses reveal suppression of the MAPK pathway, including ERK and its downstream substrates in melanoma cells exposed to MBZ+trametinib. BAK cells were exposed to 100 nM of MBZ, trametinib, or a combination of the two. Cell extracts derived at indicated times were subjected to RPPA analysis. The “pERK-targets” cluster were then plotted as time courses for phosphorylation of ERK and ELK1 (**A***,* upper panel), phosphorylation of RSK3, S6 ribosomal proteins, and eIF4E (**A**, lower panel), and cleavage of caspase-3 and PARP, as well as for levels of total cMYC (**B**).

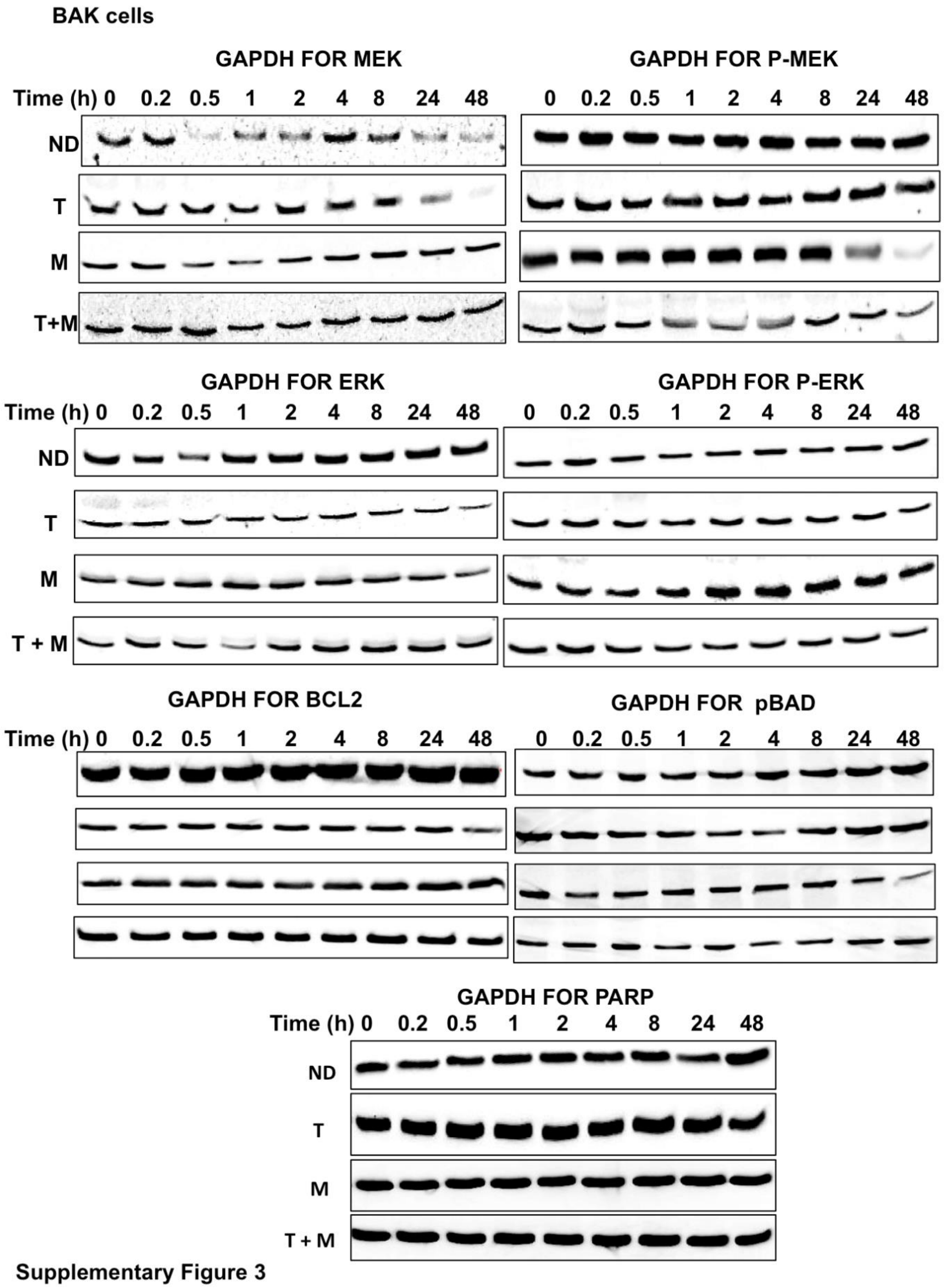

Supplementary Figure 3: GAPDH immunoblots for BAK cell extract loading controls using membranes shown in Figure 5 *B-E*.

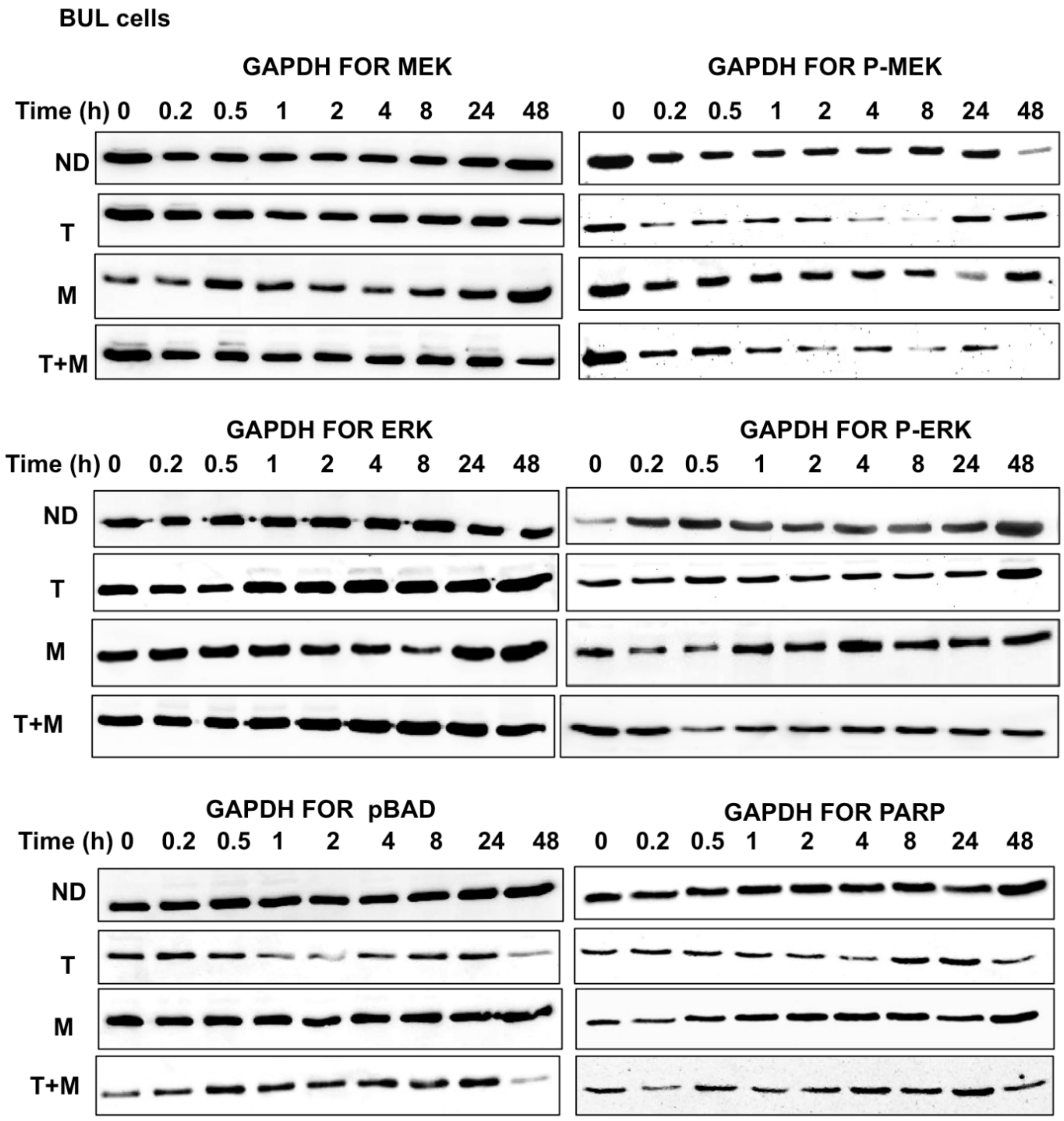

Supplementary Figure 4: GAPDH immunoblots for BUL cell extract loading controls using membranes shown in Figure 5 *F-H*.

Supplementary Table 1: Rate-based T/C and p-values of tumor xenografts analyzed from data shown Figure 4A.

	Rate-based T/C	p value
**HDT vs Vehicle**	0.73	0.26
**HDT+MBZ vs Vehicle**	0.73	0.038
**LDT vs Vehicle**	0.93	0.65
**LDT+MBZ vs Vehicle**	0.77	0.066
**MBZ vs Vehicle**	1.04	0.87
**HDT vs HDT+ MBZ**	1.02	0.95
**LDT vs LDT+ MBZ**	0.81	0.20

Supplementary Table 2: Synergy Analysis from data shown Figure 2.

BAK cells **NRAS Q61K mutation**

Combinations	Dose (nM)	Combination Index	Relationship
T+MBZ	1	0.2553855	synergistic
T+MBZ	10	0.0116237	synergistic
T+MBZ	100	0.00035	synergistic
T+MBZ	1,000	0.000168	synergistic
T+MBZ	10,000	0.000018	synergistic
T+D	1	7.841233194	antagonistic
T+D	10	0.226276739	synergistic
T+D	100	0.002946497	synergistic
T+D	1,000	0.019861052	synergistic
T+D	10,000	0.020159478	synergistic

BUL cells **NRAS Q61K mutation**

Combinations	Dose (nM)	Combination Index	Relationship
T+MBZ	1	0.00075307	synergistic
T+MBZ	10	0.00007310	synergistic
T+MBZ	100	0.00005691	synergistic
T+MBZ	1,000	0.00007690	synergistic
T+MBZ	10,000	0.01405457	synergistic
T+D	1	2.29282989	antagonistic
T+D	10	0.03043452	synergistic
T+D	100	0.02175783	synergistic
T+D	1,000	0.04063049	synergistic
T+D	10,000	0.29565493	synergistic

STU cells	BRAF V600K mutation		
**Combinations**	**Dose (nM)**	**Combination Index**	**Relationship**
T+MBZ	1	2.514318	antagonistic
T+MBZ	10	2.574866	antagonistic
T+MBZ	100	2.517441	antagonistic
T+MBZ	1,000	1.009036	additive
T+MBZ	10,000	10.067305	antagonistic
T+D	1	2.091955	antagonistic
T+D	10	2.634046	antagonistic
T+D	100	0.844587	additive
T+D	1,000	1	additive
T+D	10,000	10	antagonistic
